# New Treatment of Plaster Casts

**Published:** 1878-01

**Authors:** 


### ARTICLE III.

### *New Treatment of Plaster Casts.*

In response to the offer of a prize by the Prussian Government for a method of so treating plaster casts as that they may be washed, and at the same time that the pores of the material shall be so closed as to prevent the entrance of dirt, Dr. Ressig gives two different processes whereby the plaster is transformed into a combination, insoluble not only in water, but in soap suds.

The first method is based on the fact that sulphate of lime is changed by baryta water into sulphate of baryta, a caustic lime gradually transformed by the action of the atmosphere into a carbonate of lime, as we know from the similar action of plaster and mortar. Baryta water is prepared by slacking together in a securely corked bottle one part of crystallized hydrate of baryta with about twenty parts of rain water, thus forming a saturated solution. After it has cleared, it is sponged or poured over the plaster as long as the cast will absorb it. The cast is then dried moderately and more baryta water applied, if it still takes it in. This treatment, besides hardening the cast, gives it a beautifully white and clear appearance.

The other method is by using a silicate of potash. To obtain this, water containing about ten per cent. of caustic potash is heated to 212° F., and to this is added as much silicic acid as the potash solution will dissolve. Some clay and silicate of potash are precipitated on its cooling. Preserve this liquid in tightly stopped bottles until no signs of turbidity are seen. When about to use it, throw in a small piece of potash—one or two per cent. The solution is applied by dipping the cast, or by applying the liquid with a sponge or spray. An almost instantaneous change occurs, when the superfluous solution should be carefully washed off with soap-suds. Some practice is necessary to determine the time of exposure by this last method.

Casts thus treated are no longer, so far as their surfaces are concerned, plaster, and are very hard and durable. They are still however porous, and absorb dirt. To close the pores, dissolve one part of castile soap in ten parts of alcohol, and wash the cast with this, which will close the pores. A better way, though more expensive, is to dissolve steatite of sodium in pure spirits of wine, and warm the cast before applying. This gives an almost vitreous appearance to the cast, and produces an exceedingly beautiful appearance.

